# Poland Syndrome in a Pregnancy From an Assisted Reproductive Technology (ART) Cycle With In Vitro Maturation (IVM) and Rescue Intracytoplasmic Sperm Injection (ICSI)

**DOI:** 10.7759/cureus.56936

**Published:** 2024-03-26

**Authors:** Nikos Petrogiannis, Savvas Petrogiannis, Maria Filippa, Chalent Alexakis, Katerina Chatzimeletiou

**Affiliations:** 1 Obstetrics and Gynecology, In Vitro Fertilization Unit, Naval Hospital of Athens, Athens, GRC; 2 Medicine, In Vitro Fertilization Unit, Naval Hospital of Athens, Athens, GRC; 3 Clinical Embryology, In Vitro Fertilization Unit, Naval Hospital of Athens, Athens, GRC; 4 Obstetrics and Gynaecology, General Hospital of Lamia, Athens , GRC; 5 Genetics, Unit for Human Reproduction, 1st Department of Obstetrics and Gynaecology, Aristotle University of Thessaloniki, Thessaloniki, GRC

**Keywords:** coiled umbilical cord, in utero death, rescue intra cytoplasmic sperm injection (icsi), in vitro maturation (ivm), poland syndrome

## Abstract

Poland syndrome is a congenital anatomical anomaly, characterised by partial or total aplasia of one side of the body causing abnormalities affecting the chest, shoulder, and upper limb. The exact mechanism that leads to this syndrome is unknown, but an abnormality in the vasculature formation or interruption of the blood supply of the subscapular artery and its branches early in development may be the main cause. Depending on the underlying mechanism, the syndrome has several expressions with some hardly being detectable and others not even being compatible with life. Here, we present a case of pregnancy from an assisted reproductive technology (ART) cycle with in vitro maturation (IVM) and rescue intra cytoplasmic sperm injection (ICSI), which resulted in the in-utero death of the foetus. The subsequent necropsy revealed a variation of Poland syndrome.

## Introduction

Poland syndrome is a pathological condition that causes unequal muscle development on one side of the body. It is characterized by dysplasia of the chest wall and syndactyly on the same side [1]. More specifically, it can present with the absence or underdevelopment of the pectoralis major muscle, associated sometimes with hypoplasia of the breast, agenesis of 2nd,3rd,4th, and 5th ipsilateral costal cartilage, athelia, and ipsilateral webbing of the fingers (syndactyly) [2,3]. Affected individuals may have several presentations such as underdevelopment or absence of one nipple including the areola and/or patchy absence of hair in the axilla [2,4]. It has been observed that the affected female population might have an underdevelopment or aplasia of one breast and the underlying subcutaneous tissues.

The syndrome was named after the British surgeon Alfred Poland, who first described the condition in the 19th century [5]. Its range is estimated to be between 1:7000 and 1:100000 births with a higher frequency among males (ratio 2:1-3:1) [4]. Depending on the underlying cause, the syndrome has several expressions with some being not even detectable and others not compatible with life [6]. Poland syndrome is a sporadic event, most likely caused by interruption of the blood supply of the subscapular artery and its branches during the 6th week of development. Rare cases of Poland syndrome may have a genetic predisposition passed down in families, but no related genes have been identified [4-10]. Here, we present a case of a pregnancy from an assisted reproductive technology (ART) cycle with in vitro maturation (IVM) and rescue intra cytoplasmic sperm injection (ICSI), which resulted in an in-utero death of the foetus; the subsequent necropsy revealed a variation of Poland syndrome.

## Case presentation

A couple was referred to the Unit for Human Reproduction of the Naval Hospital of Athens for infertility treatment due to oligospermia. The 34-year-old female patient (gravida 0) underwent ovarian stimulation with gonadotrophin administration (Menopur; Ferring U.S. Headquarters, Parsippany, New Jersey) starting on day 2 of the menstrual cycle followed by antagonist administration seven days later (Cetrotide; Merck- Darmstadt, Germany). The patient was monitored regularly by ultrasound and assessment of oestradiol (E2) levels. When adequate follicular development was demonstrated (3 follicles of 17mm in diameter), human chorionic gonadotrophin (hCG) (Ovitrelle; Merck- Darmstadt, Germany) was administered to trigger final oocyte maturation. Thirty-seven hours after hCG administration, transvaginal ultrasound-guided oocyte retrieval was performed and 5 cumulous/oocyte complexes (COCs) were retrieved by flushing ovarian follicles with HTF medium (SAGE- Malov, Denmark).

**Figure 1:**
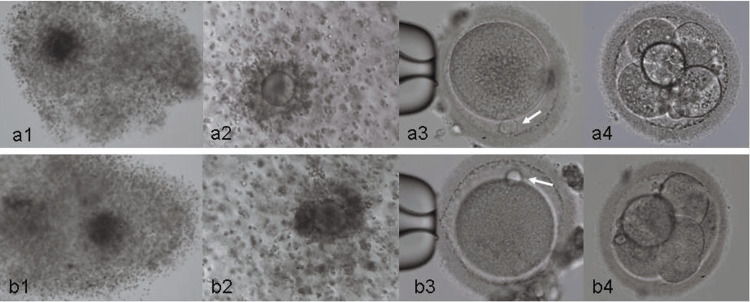
Maturation and development of the oocyte that was subjected to IVM and ICSI (a1-a4) Maturation and development of the oocyte that was subjected to IVM and ICSI led to the 6-cell embryo that was transferred to the uterus. (a1) The cumulus/ oocyte complex (COC) at egg retrieval (DAY 0) showing signs of immaturity, (a2) The COC 25 hours post-IVM (In Vitro Maturation) before denudation, (a3) The oocyte after denudation showing a 1st polar body (arrow) (mature MII oocyte), immobilised by suction with a holding pipette ready for intra cytoplasmic sperm injection (ICSI), (a4) Development of the fertilised oocyte to the 6 cell stage 95.5 hours post-ICSI (Intracytoplasmic Sperm Injection) at the time of embryo transfer. (b1-b4) Maturation and development of the oocyte that was subjected to conventional IVF and rescue ICSI led to the early morula that was transferred to the uterus. (b1) The COC at egg retrieval (DAY 0) showing signs of immaturity, (b2) The COC 19 hours post-IVF before denudation for pronuclei (PN) check, (b3) The oocyte after denudation showing no signs of fertilisation following IVF. Note the 1st polar body (arrow) indicating a mature MII oocyte which was subsequently subjected to rescue ICSI, (b4) Development of the fertilised oocyte to the early Morula stage 93.5 hours post-rescue ICSI at the time of embryo transfer.

The COCs appeared immature (Day 0) and it was decided to culture 3 COCs in LAG medium (Origio, Malov, Denmark) for 3 hours followed by in vitro maturation (IVM) medium (Origio, Malov, Denmark) for 22 hours in 5% CO2/ 5% O2 at 37°C, while the remaining 2 COCs were processed for conventional IVF by adding 500,000 motile sperm/well containing the 2 COCs. Pronuclei (PN) check 19 hours post-IVF (Day 1) revealed no signs of fertilization and rescue intracytoplasmic sperm injection (rescue ICSI) was performed. Eighteen hours post rescue ICSI (Day 2), 1 normally fertilized oocyte (2PN) was observed. Denudation of the 3 IVM COCs revealed that all 3 oocytes were at the metaphase II (MII) stage, and therefore, they were processed for ICSI (Day 1). Male pronucleus (PN) check 18 hours post ICSI (Day 2) revealed 1 normally fertilized oocyte (2PN). Both, the rescue ICSI zygote and the IVM/ICSI zygote cleaved, and 93.5-95.5 hours post-ICSI developed to the early morula stage and 6-cell stage, respectively, and were transferred to the uterus (Figure 1).

**Figure 2:**
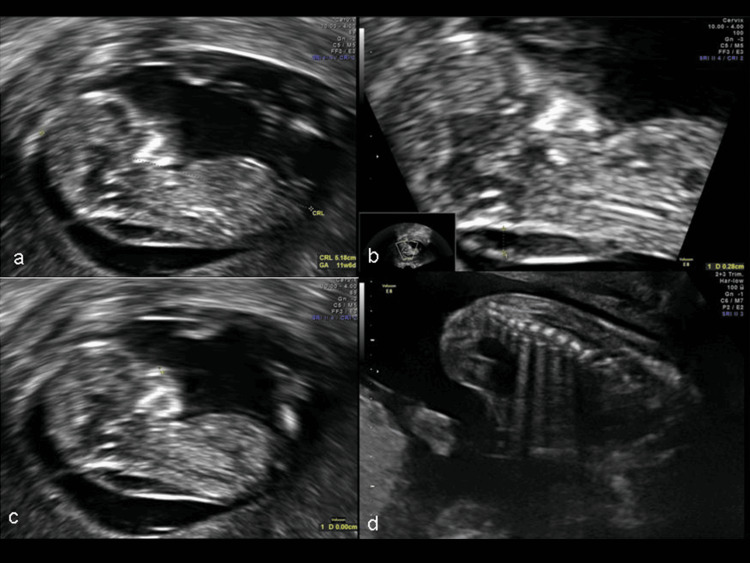
Ultrasound scan of the foetus at 11+6 weeks (a-c) and at 22+6 weeks (d). (a)Crown Rump Length (CRL), (b) nuchal translucency (NT), (c) nasal bone, (d) anatomy of ribs

Ten days after the embryo transfer, a positive serum β-hCG test was obtained (95 IU). An ultrasound scan at 6 weeks of gestation confirmed the presence of one foetal sac, and positive heart function at 7 weeks of gestation. The course of the pregnancy was normal and at 11+6 weeks, the patient had her 1st trimester ultrasound assessment (nuchal translucency (NT) measurement) along with the corresponding blood tests (PAPP-A,β-hCG). The NT was found to be 3.00mm, and along with the results of the PAPP-A (0,549 MoM) and β-hCG (0,484 MoM), the probability of a chromosomal abnormality was high. Following genetic counseling, the patient decided to undergo amniocentesis at 18 weeks gestation. The amniotic fluid sample was analysed by QF-PCR, which showed the absence of aneuploidy for chromosomes 13,18,21 and the sex chromosomes. A pair of X chromosomes was detected, indicating a female embryo. Furthermore, the CytoScan Protocol was applied to analyse the whole genome for chromosomal abnormalities and submicroscopic gains and losses that also detected no abnormalities. The pregnancy continued normally and the patient had her 2nd trimester ultrasound scan at 22+6 weeks (Figure 2). A single umbilical artery was discovered, while the foetal anatomy, growth, and movements appeared normal in the second-trimester routine ultrasound scan. At 29 weeks, due to reduced fetal movements, an ultrasound scan was performed. The development of the foetus was within the normal range, all Doppler measurements were normal, and the foetal weight was 1,284g. At 29+5 weeks, the patient complained again of absent foetal movements, and thus, another ultrasound scan was carried out, which revealed no cardiac activity.

**Figure 3:**
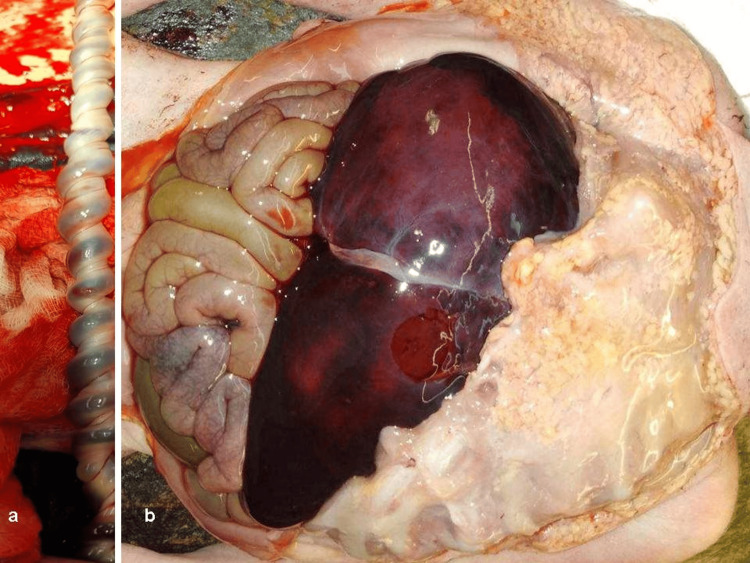
(a) The highly coiled umbilical cord and (b) absence of the right rib cage

A cesarean section was subsequently performed and a 1018g female fetus was delivered. A notable feature was that the umbilical cord was highly coiled, looking like a telephone cord. The foetus was sent for a necropsy and the following findings were discovered: a) external features: misshapen face, protruding nose, micrognathism, low ear adhesion, short cervix, protruding shoulders, hollowing of the right back side, missing of the right hemithorax, and missing of the right nipple, b) internal organs: rib agenesis of the right hemithorax, brain weight/thymus weight >normal (indication of fetal distress), bilateral lung hypoplasia, hypoplasia of the right adrenal gland, and a thin single artery umbilical cord with 0.35 coils/cm (total length 33cm) (Figure 3).

## Discussion

Poland syndrome is a rare disease that is more frequently observed in males than females [7]. The condition is defined as a hypoplasia or lack of fat muscles, bones, and mammary glands, including the nipple and the areola on one side of the chest. In addition, it affects the hair growth of the armpit and the development of the joint of the upper limb (syndactyly or symphalangism) [8]. The absence of the sternum of the pectoralis major muscle represents a minimal expression of this condition. The right side of the chest is affected more frequently than the left side [8].

The exact mechanism that leads to this syndrome is unknown. It is assumed that the spectrum of aplasia of pectoralis muscles, the athelia, and aplasia of costal ribs are consequences of interruption of the blood supply of the subscapular artery and its branches during the 6th week of development [4, 9-10]. It is known that the subscapular artery or the axillary artery gives branches to key arteries that supply the thoracic wall like the medial thoracic branches, intercostal arteries, and thoracic artery. Due to the complexity of the syndrome and the unknown mechanism of pathogenesis, the syndrome has several aspects of presentation, some more and some less serious. It is believed that Poland syndrome is a sporadic event and rarely an inherited one. In the literature, there are rare instances where one member of a family is identified with this condition, with no cases in the immediate or extended family. As a consequence, it is suggested that an abnormality in the vasculature formation may be the main cause of this sequence [4, 9-10]. Although it is not easy to detect Poland Syndrome prenatally by ultrasound monitoring, some of its most common anatomical abnormalities (widening of coronal sutures, absence of the ossification of cranial and pubic bones, absence/hypoplasia of nasal bone, and hypoplasia of the thoracic cage) found in affected foetuses may be detected prenatally by ultrasound scan [11].

The foetus in our case had an increased nuchal translucency and a single umbilical artery, but no other anomalies associated with the syndrome were detected prenatally. These are sporadic findings that can be correlated with a variety of syndromes and are not specific to the Poland syndrome. It was only after necropsy that several features of Poland syndrome were identified, as previously described in the literature [1-10], including protruding shoulders, hollowing of the right back side, rib agenesis of the right hemithorax, missing right hemithorax and missing right nipple, bilateral lung hypoplasia, and hypoplasia of the right adrenal gland. The brain weight and thymus weight were more than normal, indicating foetal distress, and the highly coiled, thin, single artery umbilical cord, with 0.35 coils/cm may suggest a risk for blood supply disruption.

Umbilical cord anomalies present risk factors for stillbirth [12-14]. Umbilical cords with right twists have been highly associated with chronic fetal vascular obstruction and in-utero death, compared to cords with left twists. Hypercoiled cords, with patterns of significant indentation or pinching of the cord diameter, are correlated with histological evidence of chronic fetal vascular obstruction and stillbirth [13]. Extensive umbilical cord hypercoiling (displaying an umbilical coiling index of >1.0 coils/cm), could be a pathogenetic factor for the development of massive perivillous fibrin deposition, and its recognition in the second trimester by ultrasound may have a predictive value for the identification of fetal hypotrophy, necessitating intensified foetal monitoring to avoid adverse foetal outcome [14].

The pregnancy in the current case was derived from an ART cycle that was complicated by the fact that no mature cumulus/oocyte complexes were collected on the day of retrieval (Day 0) and IVM and rescue ICSI were applied, leading to the 2 fertilised oocytes, that cleaved and were transferred on day 5 post-oocyte retrieval to the uterus. Although it is uncertain which one of the two embryos implanted successfully and led to this singleton pregnancy, it should be noted that the embryo derived from IVM was slower in cleavage compared to the embryo derived from failed IVF and rescue ICSI. For patients with low numbers of mature MII oocytes at egg retrieval, rescue IVM may be an attractive approach to provide additional competent oocytes and embryos for transfer, maximizing the chances for pregnancy [15]. Although IVM appears to be associated with only a small amount of epigenetic variation in cord blood and placental tissue [16], it has been previously linked to early pregnancy loss [17]. However, in women with a serum AMH ≥10 ng/ml, IVM and standard ovarian stimulation cycles resulted in comparable reproductive outcomes [18]. The serum AMH of the female patient in the present report was 2.45ng/ml.

Fertilization and blastulation rates have been shown to be significantly higher for oocytes that were mature on the day of egg retrieval (MII-Day 0) compared to delayed-matured oocytes (MII-Day1). However, no significant difference was observed for embryo euploidy between MII-Day0 and MII-Day1 oocytes [19]. Rescue ICSI offers a unique second opportunity for previously failed fertilised oocytes. Lower live birth rates have been reported for rescue ICSI following both fresh cleavage (12.3%) and blastocyst (26.3%) stage transfers compared to frozen/warmed blastocyst transfers (46.7%), suggesting that resynchronization of embryo developmental stage with the endometrium can optimize rescue ICSI outcomes [20].

## Conclusions

This case report describes a pregnancy from an ART cycle with IVM and Rescue ICSI, which resulted in an in-utero death of the foetus at 29+5 weeks, and the subsequent necropsy revealed a variation of Poland syndrome. Prenatal ultrasound monitoring of the foetus did not detect any features of the syndrome, but an increased nuchal translucency was evident. Diagnosis of this condition in utero is very important to inform parents of the survival rates, prognosis, and morbidity rates, and in the event of pregnancy termination, necropsy may be mandatory to ensure correct diagnosis. Although Poland syndrome is rarely an inherited condition, it usually results from an abnormality in the vasculature formation or interruption of the blood supply of the subscapular artery and its branches early in development. Whole exome sequencing may be useful in identifying mutations that may play a role in the aetiology of the syndrome. Larger scale studies are needed to elucidate any potential impact of IVM or rescue ICSI on the well-being of the foetus.
